# Image Mapping Accuracy Evaluation Using UAV with Standalone, Differential (RTK), and PPP GNSS Positioning Techniques in an Abandoned Mine Site

**DOI:** 10.3390/s23135858

**Published:** 2023-06-24

**Authors:** Hanjin Kim, Chang-Uk Hyun, Hyeong-Dong Park, Jongmun Cha

**Affiliations:** 1 Department of Energy Systems Engineering, Seoul National University, Seoul 08826, Republic of Korea; sighchris@snu.ac.kr; 2 Department of Energy and Mineral Resources Engineering, Dong-A University, Busan 49315, Republic of Korea; jmcha@dau.ac.kr; 3 Department of Energy Resources Engineering, Seoul National University, Seoul 08826, Republic of Korea; hpark@snu.ac.kr

**Keywords:** mine tailings, abandoned mine site, real-time kinematic (RTK) GNSS, post-mission precision point positioning (PPP) processing, UAV image mapping

## Abstract

Global navigation satellite systems (GNSSs) provide a common positioning method that utilizes satellite signals to determine the spatial location of a receiver. However, there are several error factors in standalone GNSS positioning due to instrumental, procedural, and environmental factors that arise during the signal transmission process, and the final positioning error can be up to several meters or greater in length. Thus, real-time kinematic (RTK) correction and post-mission precise point positioning (PPP) processing technologies are proposed to improve accuracy and accomplish precise position measurements. To evaluate the geolocation accuracy of mosaicked UAV images of an abandoned mine site, we compared each orthomosaic image and digital elevation model obtained using standalone GNSS positioning, differential (RTK) GNSS positioning, and post-mission PPP processing techniques. In the three types of error evaluation measure (i.e., relative camera location error, ground control points-based absolute image mapping error, and volumetric difference of mine tailings), we found that the RTK GNSS positioning method obtained the best performance in terms of the relative camera location error and the absolute image mapping error evaluations, and the PPP post-processing correction effectively reduced the error (69.5% of the average total relative camera location error and 59.3% of the average total absolute image mapping error) relative to the standalone GNSS positioning method. Although differential (RTK) GNSS positioning is widely used in positioning applications that require very high accuracy, post-mission PPP processing can also be used in various fields in which it is either not feasible to operate expensive equipment to receive RTK GNSS signals or network RTK services are unavailable.

## 1. Introduction

Global navigation satellite systems (GNSSs) combine multiple satellite signals to realize the position of a receiver. Transit, which is a satellite-based survey and navigation system that was used by the United States Navy in the 1950s [1], was the first GNSS. More recently a defense-related GNSS was developed by combining early-stage space-based navigation systems, i.e., Timation and 621B systems, which are the current Global Positioning System’s (GPS) prototypes [2]. There are several other existing GNSSs that are commonly used for positioning purposes without corrections, including GPS in the United States, Galileo in Europe, the Global Navigation Satellite System in Russia, and BeiDou in China. Differential GNSS (DGNSS) with reference receivers were also previously developed, and satellite-based augmentation systems (SBASs) that use a geostationary satellite to provide spatial corrections for a wide area (e.g., the European Geostationary Navigation Overlay System (EGNOS), the Multi-Functional Satellite Augmentation System (MSAS), GPS-aided GEO Augmented Navigation (GAGAN), the System for Differential Correction and Monitoring (SDCM), and the Wide Area Augmentation System (WAAS)), which are DGNSS-derived wide-area information navigation systems, were previously introduced [3,4].

The first real-time kinematic (RTK) GNSS positioning system calibrated in centimeters was developed in 1994, and this system implemented techniques that addressed the various error factors of existing low-precision GNSSs. The precise point positioning (PPP) post-processing correction technique was developed in 1995 to address the limitations associated with RTK GNSS positioning’s distance or infrastructure constraints [5], and both positioning methods are currently used to realize accurate and precise positioning technologies. Such technologies can be implemented in various types of equipment; currently, they are mounted onto unmanned aerial vehicles (UAVs) and used to record a built-in receiver’s precise location information when imaging specific objects or terrain.

In addition, GNSS-based positioning technology is used in various industries, e.g., mining. Mining sites utilize UAVs to determine the precise location information regarding mine operations in various mining areas, e.g., surface, underground, and abandoned mines [6]. Here, various sensors are attached to UAVs, including digital cameras, spectral cameras, light detection and ranging systems, and geophysical sensors, to perform various tasks, e.g., three-dimensional (3D) reconstruction and ecological environmental monitoring near mining sites [7]. Such systems are also used to investigate various hazards, e.g., subsidence near abandoned mines [6]. Precise positioning methods, such as RTK GNSS positioning or post-mission PPP processing, are indispensable in terms of effectively achieving these goals.

Numerous studies separately assessed the performance of RTK GNSS positioning and post-mission PPP processing. For example, Lee and Ge [8] evaluated the performance of standalone GNSS and RTK GNSS positioning under various urban and vegetation conditions, and Peppa et al. [9] investigated how the performance evaluation of planes and vertical errors is affected by whether UAVs include a RTK GNSS positioning function. Czyża et al. [10] demonstrated the accuracy assessment in drone pose estimation with RTK positioning as a means of determining drones’ applicability to surveying operations. In addition, Alkan et al. [11] evaluated the accuracy of the Canadian Spatial Reference System, which is a PPP processing method, and the Trimble CenterPoint RTX, which is a real-time global solution method. Qafisheh et al. [12] utilized a prediction model to correct the time latency of a GNSS to obtain a better performance of PPP.

Recently, Ocalan et al. [13] investigated the accuracy of PPP and PPP-AR (PPP positioning with ambiguity resolution) by comparing RTK UAV flight data. However, to the best of our knowledge, no previous study attempted to compare the performance, i.e., the positioning accuracy, of RTK GNSS positioning and post-mission PPP processing techniques from an image mapping accuracy perspective, because PPP processing is an upcoming GNSS positioning technique.

Even though RTK GNSS is the most reliable technology available for positioning, it can be difficult to use if related infrastructure (e.g., nearby reference stations or broadcasting for network RTK correction) is limited. In addition, acquiring positioning equipment with RTK receivers for use at industrial sites can be prohibitively expensive, thereby making it difficult to carry out small-scale studies and experiments [14]. Thus, in this study, we evaluated the relative applicability of post-mission PPP processing, which can be utilized as an alternative to overcome the low accuracy of standalone GNSS in UAV investigations, compared to network RTK GNSS positioning at an abandoned mine site.

## 2. Materials and Methods

### 2.1. Study Area

**Figure 1:**
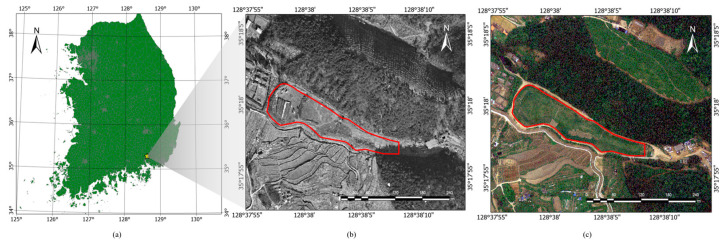
(**a**) Location of study site (Guryong mine, Changwon-si, Gyeongsangnam-do, Republic of Korea, which was abandoned in 1988). (**b**) Aerial image acquired before mine reclamation work in December 2001 (image from National Geographic Information Institute, Republic of Korea (http://map.ngii.go.kr) accessed on 2 June 2023). (**c**) Aerial image taken after mine reclamation work in May 2010 (image from National Geographic Information Institute, Republic of Korea (http://map.ngii.go.kr) (accessed on 15 July 2022), overlayed by boundary of mine tailings (red polygon)).

The study area was the Guryong mine, which constituted an abandoned copper mine located in Changwon-si, Gyeongsangnam-do, the Republic of Korea. This mine was abandoned in 1988; however, while active, it produced approximately 13,000 tons of metal. The area around the Guryong mine was underlain by andesite, which is the host rock for copper deposit, as well as a pyrite-rich tailings impoundment that was operational from 1945 to 1971 [15]. Sulfide-rich tailings, e.g., pyrite, are potential sources of acidity due to the oxidation of sulfide minerals [16], and major pollution can result from the heavy metals of leachates found in mine tailings heaps and waste rock piles [17]. Runoff from the tailings can flow into nearby streams and farmlands [15]. A mine reclamation project was conducted between 2006 and 2007. The location of the Guryong mine site and the aerial images acquired before and after the reclamation work are shown in Figure 1.

### 2.2. Applied Positioning Method

#### 2.2.1. Standalone GNSS Positioning

**Figure 2:**
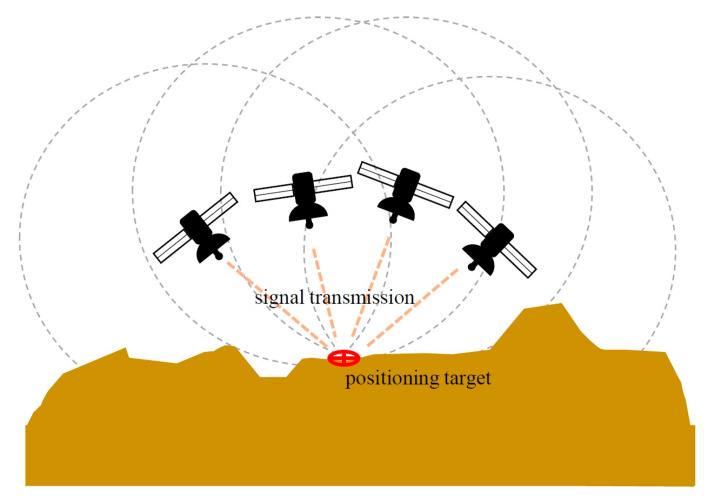
Basic principle of standalone GNSS positioning. Each satellite transmits a signal to a built-in receiver to determine distance between satellite and target object. By collecting signals from more than three satellites, approximate spatial position of target equipment can be calculated.

The standalone GNSS positioning principle was based on determining the distance between a receiver and a satellite. Here, an electromagnetic wave signal allowed the indirect distance calculation between the satellite and the receiver based on the signal arrival time and wavelength, and 3D (x, y, z) positioning was realized by exchanging signal differences between four or more satellites in order to correct the receiver time error [18] (Figure 2).

Standalone GNSS positioning was initially used for military operations [2]; however, it is currently used for various purposes, e.g., traffic navigation [19], and it is the most common and accessible positioning tool because a GNSS receiver can be embedded into personal communication equipment [20].

Despite its common use, standalone GNSS positioning can suffer relatively large positioning errors due to various error factors that occur during signal transmission, such as clock-related errors (e.g., satellite clock errors and receiver clock errors), satellite orbit errors, ionospheric delays, tropospheric delays, receiver noise, and multipath issues [21]. Therefore, high-precision GNSS positioning can be accomplished by compensating for these errors using differential GNSS positioning or post-mission PPP processing techniques.

#### 2.2.2. Differential (RTK) GNSS Positioning

**Figure 3:**
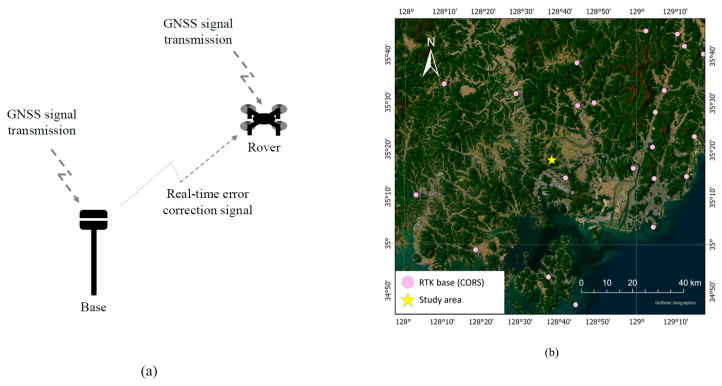
(**a**) Basic principle of RTK GNSS positioning with use of a UAV. System consists of a base and a rover. Base has an accurate reference position with correction information while receiving GNSS positioning signal, and rover hovers around measuring area while receiving GNSS positioning signal. (**b**) A Continuously Operating Reference Station (CORS), which can serve as base of network RTK GNSS positioning. Nearest station (Changwon) is about 8.95 km away from study area.

With standalone GNSS positioning, there is a certain degree of inaccuracy because phase and code ranges are superimposed or falsified by the aforementioned different error sources, and RTK GNSS positioning attempts to correct this problem by building observation differences and performing ambiguity fixing using integers. RTK GNSS positioning involved a base station and a rover, as shown in Figure 3a. The local error of the base was analyzed in real time by aggregating receiving signals and the known location, and the correction value was applied to the GNSS signal received by the rover to determine the precise final position. In this study, we utilized more easily accessible network RTK GNSS positioning, rather than installing a base in the field; thus, the RTK GNSS positioning described in this paper could be considered a network RTK GNSS positioning technique.

Generally, receiving only L1 carrier signals did not remove the delay effect that occurs in the ionosphere, whereas DGNSS or RTK GNSS receivers receiving both L1 and L2 in different frequency bands could correct errors by combining carrier signals [20]. In addition, most GNSS errors, e.g., orbital errors, satellite clock errors, and bias, could also be calibrated [22].

The most significant advantage of RTK GNSS positioning was its high accuracy. When used properly, RTK GNSS positioning was an effective measurement method, with errors of approximately 1 cm in terms of positioning accuracy [22]. However, RTK GNSS positioning posed several limitations in practical use. Firstly, a RTK GNSS receiver incurred a cost issue in places where virtual reference station (VRS) service is unavailable. Equipment with RTK GNSS that was used as the base and rover typically costs more than standalone GNSS receiver equipment. In addition, atmospheric conditions had an impact on errors in RTK GNSS positioning. Most importantly, the accuracy of RTK GNSS was affected as the base–rover distance increased. At a small base–rover distance, the correlation between ionospheric and tropospheric errors was very high, thereby making it easy to correct these errors. However, as the distance between the base and rover increased, the error correlation decreased, and sufficient accuracy cannot be guaranteed through signal correction [23]. Generally, accuracy was valid within a range of 30–50 km.

RTK GNSS positioning is currently utilized in various fields, including environmental monitoring, coastline mapping, and highway geometry [24,25,26]. In addition, many recent aerial imaging UAVs are outfitted with RTK GNSS receivers [27].

#### 2.2.3. Post-Mission PPP Processing

**Figure 4:**
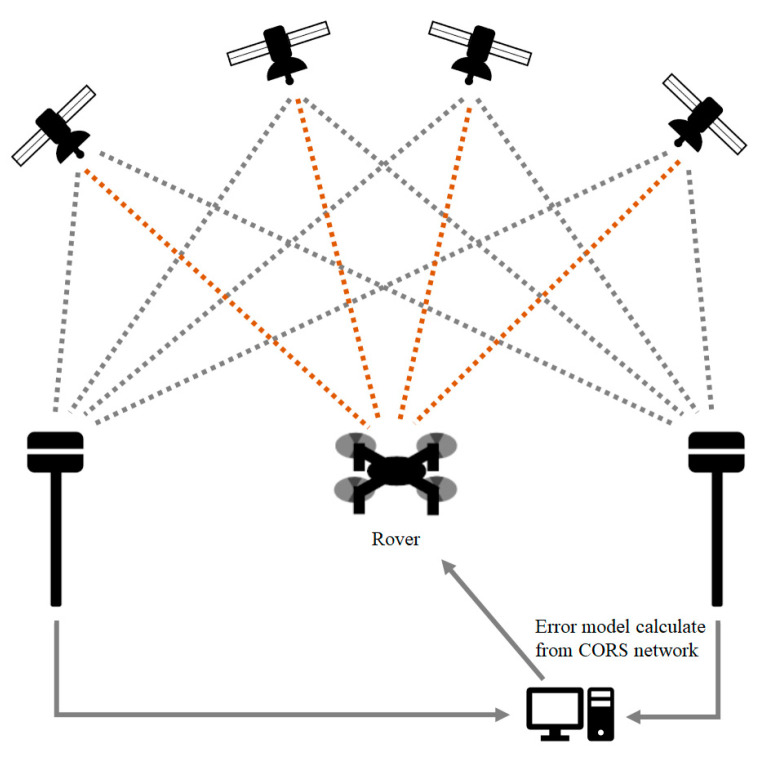
Basic principle of post-mission PPP processing positioning. After measuring GNSS receivers’ positions around study area, a modeled error is given to revise a target position.

In contrast to RTK GNSS positioning, the post-mission PPP processing method produced a GNSS error model based on fixed core stations (e.g., continuously operating reference stations, CORS) and applied these modelled range errors (Figure 4). In this study, standalone GNSS positions were tracked at each UAV image acquisition, and post-mission PPP processing using precise global clock and orbit corrections was then carried out based on the stored standalone GNSS positioning data (e.g., [28]). Generally, unlike RTK GNSS positioning, which performs real-time correction, the PPP technique incorporated into this study employs the post-processing method [22].

It should be noted that there was no restriction on the distance between a CORS and the measurement location when utilizing post-mission PPP processing [29], and the cost advantage is greater than that of RTK GNSS positioning because no additional local infrastructure was required. However, since it was a predicted error model, it was a precision error in centimeters and decimeters, which was inferior to the RTK GNSS positioning method. In addition, the convergence time was 5–30 min, which was greater than the time required via RTK GNSS positioning [22,30].

In this study, MakeItAccurate (Klau Geomatics Pty Ltd., New South Wales, Australia), which is a cloud-based post-mission PPP processing solution, was employed to apply post-mission PPP processing to UAV images obtained using camera locations from standalone GNSS positioning. This solution utilized both clock and ephemeris corrections from the satellite control stations within 15 min of acquiring the positioning data [31].

Post-mission PPP processing is used in many fields where more accurate positioning than standalone GNSS is required, including structural displacement detection and disaster monitoring [32,33]. However, post-mission PPP processing receives less attention in UAV research because current UAVs are equipped with RTK GNSS receivers. We browsed Scopus (https://www.scopus.com/ accessed on 20 June 2023) [34] to search for articles containing “RTK positioning” and “UAV” or “drone” and found 285 articles as of 6 January 2023. In comparison, we identified 42 articles containing the keywords “PPP positioning” and “UAV” or “drone” as of 6 January 2023. RTK GNSS positioning and UAVs were combined as a research topic in 2004, whereas PPP processing was introduced to support UAV research from 2010 onward. This literature review confirms that PPP positioning is an upcoming GNSS positioning technique, which is also confirmed by the increasing number of technical manuscripts dealing with this technique.

### 2.3. UAV Data Acquisition

**Figure 5:**
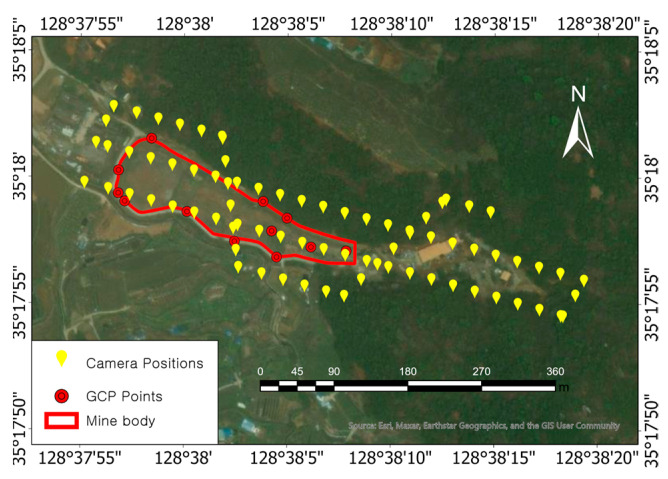
We measured 12 GCPs (red circles), and UAV-acquired aerial images from 77 positions are shown as yellow symbols (base map obtained using ArcGIS Pro (ESRI, Redlands, CA, USA)).

The data considered in this study were acquired as follows: the UAV mission planner software DJI GS Pro 2.1.9 was used to automatically establish a flight route by referencing a base map in a single data-acquisition routine, and a DJI Phantom 4 RTK UAV flew to the designated position to acquire images using the automatic flight mode. Here, the imaging procedure captured a total of 77 images, with 75% forward and 70% side overlaps, by considering the sloped study area (Figure 5).

In the RTK GNSS positioning mode, the DJI Phantom 4 RTK UAV used the network RTK GNSS positioning function in a functional network environment; however, it only received standalone GNSS signals without RTK correction if this mode was disabled. The detailed specifications of this UAV are presented in Table 1. On 11 March 2022, the RTK GNSS positioning mode and the standalone GNSS mode flights and image acquisitions were each performed three times. The nearest VRS station (Changwon) was located about 8.95 km away from the study area (Figure 3b). Thus, we acquired a total of six datasets. Three PPP post-processing images were generated based on the datasets of images captured in standalone GNSS mode, thereby resulting in a total of nine datasets.

### 2.4. UAV Image Processing

The Metashape 1.8.1 (Agisoft LLC, St. Petersburg, Russia) software is a photogrammetry pipeline tool that converts two-dimensional (2D) images, e.g., conventional photographs, images acquired via UAVs, and aerial images, into functional 3D models and orthomosaic images. Using Metashape, images taken in a specific area were merged to express topographic changes in elevation using the triangulation principle, and a digital elevation model (DEM) and orthomosaic images with co-ordinate information were created by synthesizing the location information stored in the images.

**Figure 6:**
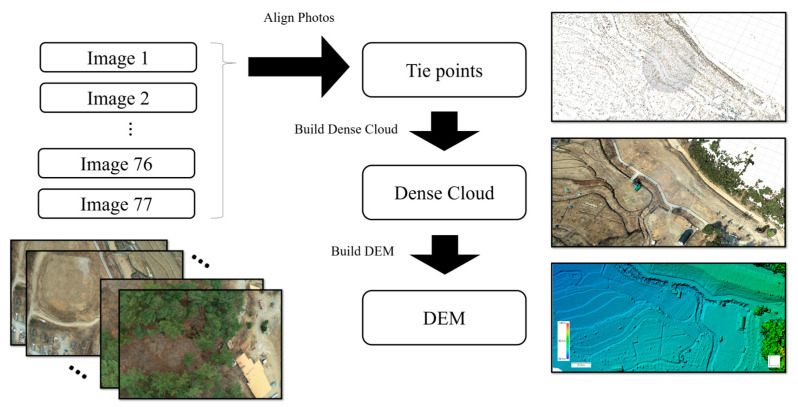
UAV image processing using Metashape to convert a dense point cloud into a DEM.

In Metashape, a group of images for a specific area with location data obtained from GNSS signal reception was designated as a chunk, and when the co-ordinate system was configured for an image chunk with metadata, Metashape automatically performeds adjustments using collinearity equations and aerotriangulation. The tie point was determined by measuring the imaging geometry of the overlapping region between images. Here, the alignment accuracy was set to medium to compromise the spatial resolution and the processing time, and the key point limit, which was the maximum number of feature points located in the overlapping area, was set to 40,000. Next, a depth map and a dense point cloud were constructed using the internal and external topographic directions, and the height value of the point cloud was interpolated based on the pixel size. As a result, an orthomosaic image, which is an image that appears to be viewed from the vertically upward direction, was generated using the color of the highest point per pixel, and a DEM was constructed using the height value of the highest point per pixel (Figure 6) [36].

### 2.5. Ground Control Point Measurement

The actual reference position and the corresponding reference position in the orthomosaic image were compared using ground control points (GCPs) to determine the accuracy of the position information based on the image mosaicking result. GCPs are widely used to accurately measure the location of multiple marked points in the field, using various equipment, e.g., a RTK GNSS receiver, to improve accuracy when matching UAV images via triangulation; the images are then georeferenced using the exact reference location of the area using geographic information system (GIS) software [37,38]. GCPs are also used in statistical analyses to compare root–mean–square errors (RMSE) to evaluate the accuracy of UAV image processing outputs, e.g., DEMs and orthomosaic images [39].

The position accuracy assessment method for orthomosaic images was used in this study, and high-precision reference positions, i.e., GCPs, were selected as clearly identifiable locations in the obtained UAV images (e.g., at the edge of a ground structure) and measured at several points near to the mine tailings area (a potential pollution source) using the RTK GNSS equipment. A field measurement was conducted in February 2022, which used the network RTK GNSS positioning method and an Emlid Reach RS2 RTK GNSS receiver. The absolute positions of the measured points served as reference measures to evaluate the orthomosaic image product’s location accuracy. The detailed specifications of the network RTK GNSS receiver are shown in Table 2.

### 2.6. Accuracy Evaluation

#### 2.6.1. Relative Camera Location Accuracy Assessment

Among the various positioning error evaluation methods, a simple method involved comparing the camera location errors estimated using the image processing procedure. Essentially, each UAV image contained metadata about a camera’s position in the WGS84 geographic coordinate system at the time of acquisition, including the positioning data from the network RTK GNSS, standalone GNSS, and post-mission PPP processing. When comparing the original camera location of the image to the inferred camera location, i.e., the result of an inverse calculation of where the camera should have been located to acquire the actual image, this method computed an orthogonal co-ordinate system’s (Universal Transverse Mercator (UTM) zone 52N) X, Y, and Z errors, as well as the X–Y plane error and the total error in three dimensions [41,42], using the following equations:

(1) $$X\;\mathrm{error}=\sqrt{\sum_{i=1}^{n}{\left(X_{i_{est}}-X_{i_{init}}\right)}^{2}}$$

(2) $$Y\;\mathrm{error}=\sqrt{\sum_{i=1}^{n}{\left(Y_{i_{est}}-Y_{i_{init}}\right)}^{2}}$$

(3) $$Z\;\mathrm{error}=\sqrt{\sum_{i=1}^{n}{\left(Z_{i_{est}}-Z_{i_{init}}\right)}^{2}}$$

(4) $$X–Y\;\mathrm{error}=\sqrt{\sum_{i=1}^{n}{\left(X_{i_{est}}-X_{i_{init}}\right)}^{2}+{\left(Y_{i_{est}}-Y_{i_{init}}\right)}^{2}}$$

(5) $$\mathrm{Total}\;\mathrm{error}=\sqrt{\sum_{i=1}^{n}{\left(X_{i_{est}}-X_{i_{init}}\right)}^{2}+{\left(Y_{i_{est}}-Y_{i_{init}}\right)}^{2}+{\left(Z_{i_{est}}-Z_{i_{init}}\right)}^{2}}$$

where $X_{i_{est}}$ is the estimated value of the *X* coordinate for the *i*-th camera location, $X_{i_{init}}$ is the initial value of the *X* coordinate for the *i*-th camera location, $Y_{i_{est}}$ is the estimated value of the *Y* coordinate for the *i*-th camera location, $Y_{i_{init}}$ is the initial value of the *Y* coordinate for the *i*-th camera location, $Z_{i_{est}}$ is the estimated value of the *Z* coordinate for the *i*-th camera location, $Z_{i_{init}}$ is the initial value of the *Z* coordinate for the *i*-th camera location, and *n* is the number of camera locations.

#### 2.6.2. GCP-Based Mapping Accuracy Assessment

Installing the GCPs using the above process (Section 2.5) produced an accurate absolute reference position, and the co-ordinates for each corresponding GCP in the orthomosaic image contained position information constructed via the matched UAV image, with little difference found between it and the actual reference location if the positioning accuracy of the matched image was sufficient. The actual network RTK GNSS-based reference position was converted into X, Y, and Z to replace $X_{i_{init}}$, $Y_{i_{init}}$, and $Z_{i_{init}}$ of Equations (1)–(5) on the Cartesian co-ordinate system (i.e., UTM zone 52N), making it possible to calculate the location errors on the same co-ordinate system of the position, while also replacing $X_{i_{est}}$, $Y_{i_{est}}$, and $Z_{i_{est}}$ of Equations (1)–(5) in the orthomosaic image and DEM. Next, X, Y, and Z errors, along with the X–Y plane phase error and the total error in three dimensions, were computed using Equations (1)–(5).

#### 2.6.3. Comparison of Areal and Volumetric Differences of Mine Tailings

**Figure 7:**
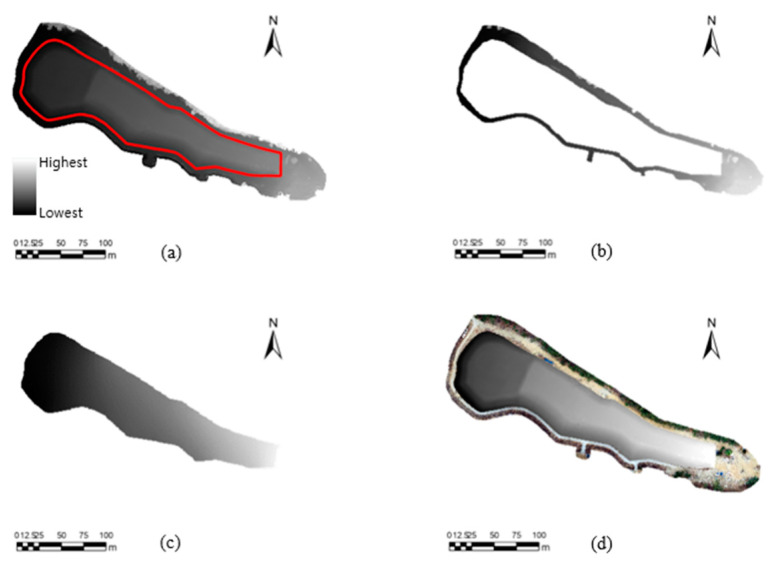
Layer images (**a**–**d**) show each step of volumetric difference assessment using DEMs obtained from three GNSS positioning techniques. In DEM of study area shown in (**a**), we defined mine tailings area with a convex boundary (red polygon). After removal of mine tailings from DEM (**b**), we interpolated boundary value to obtain a DEM of underlying surface of mine tailings. (**c**) shows DEM of underlying surface, and (**d**) shows final volumetric model of mine tailings over underlying surface, which was calculated by subtracting (**c**) from (**a**).

The volume difference of the mine tailings was evaluated to determine whether the repeatedly obtained UAV images were correctly positioned and exhibited stable positions. Here, the tailings were assumed to be loaded onto the surface parallel to the boundary of the tailings; thus, the volume of the tailings above the bottom surface of the study area must be measured to precisely evaluate the potential pollution sources. After visualizing the tailings area layer using ArcGIS Pro (ESRI, Redlands, CA, USA) (Figure 7a), the bottom surface was interpolated using the boundary height (Figure 7b) of the mine tailings’ DEM (Figure 7c) and subtracted from the existing tailing area (Figure 7a), yielding the height information of the hill structure (Figure 7d). The 2D areas (i.e., the area projected onto a flat surface), surface areas, and volumes of the mine tailings were calculated using ArcGIS Pro’s area, surface area, and volume evaluation algorithms. It should be noted that there were no measured reference 2D area, surface area, and volume data for the mine tailings; thus, the errors were calculated by determining the differences between the 2D areas, the surface areas, and the volumes from the case of the first experiment, which used the network RTK GNSS positioning, and the remaining eight cases.

## 3. Results

### 3.1. UAV Data Acquisition and Image Processing

**Figure 8:**
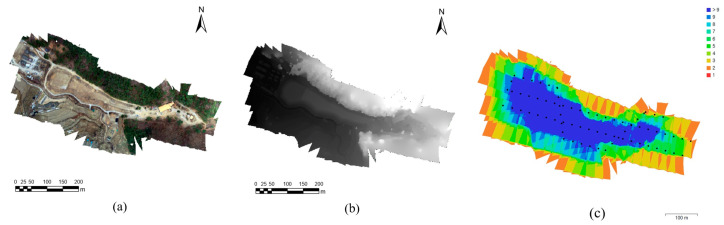
UAV image orthomosaicking results: (**a**) example of an orthomosaic image with network RTK GNSS positioning; (**b**) example of a DEM; (**c**) example of number of overlapped images in mosaicking process of study area.

Each of the nine measurements yielded nine orthomosaic images (Figure 8a shows an example of an orthomosaic image obtained using the images acquired from the first UAV flight with the network RTK GNSS positioning) and nine DEMs (Figure 8b). Figure 8c depicts the extent of overlapping in the images obtained in the study area; sufficient overlap, i.e., 75% forward and 70% side overlap, as denoted in the Materials and Methods section, enables precise 3D topographic modeling. The numbers of overlapped pictures per zone were counted, and more precise results were obtained toward the center of the zone, where the tailings area (the target area shown in Figure 5) is located. The specific flight information is given in Table 3.

A total of 9 orthomosaic images were produced using 73 images, excluding 4 images of forest areas located in the southeastern region of the study area that were located outside of the mine tailings. It should be noted that the spatial resolution of the nine orthomosaic images varied from 2.64 to 2.68 cm.

### 3.2. GCP Measurement

**Figure 9:**
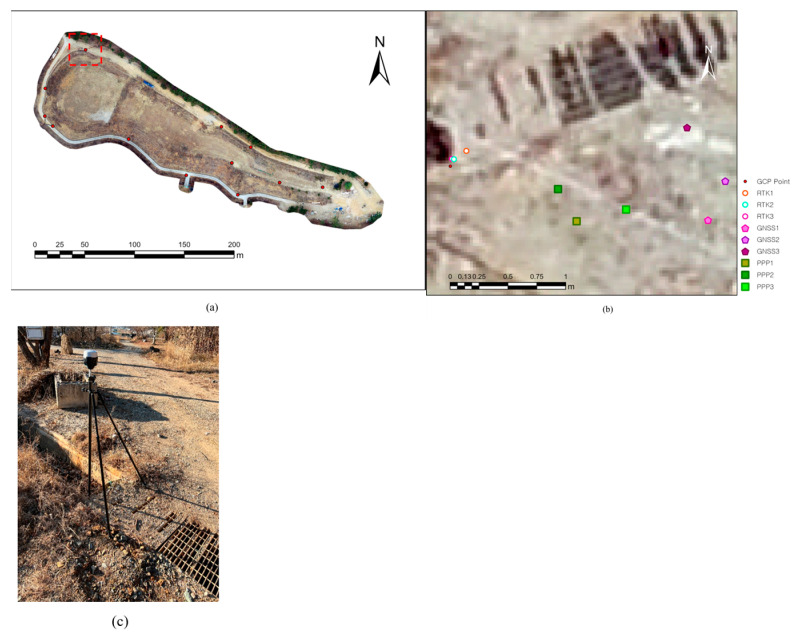
GCP measurement results: (**a**) positioning of 12 GCPs (red circles), and (**b**) positioning of corresponding GCPs in nine cases of orthomosaic images to a reference GCP (GCP No. 12 (in red rectangle (**a**)), which is measured as (**c**)).

Table 4 shows that absolute reference positions were established using twelve GCPs for clearly identifiable locations in the orthomosaic images, nine GCPs around the mine tailings boundary, and three GCPs on the mine tailings, as shown in Figure 9a, in which the network RTK GNSS equipment is used (Figure 9c). To eliminate as much random error as possible, the network RTK GNSS positioning measurements were averaged by measuring the same location five times.

### 3.3. Accuracy Assessment

#### 3.3.1. Estimation of Relative Camera Location Error

As a result of UAV image processing, information regarding nine relative camera location errors was obtained through nine image data alignments (Table 5). For the three network RTK GNSS measurements, the relative camera location errors were less than 3 cm, the images obtained using standalone GNSS positioning had errors greater than 1 m, and the post-mission PPP processing result had a relatively large deviation of approximately 27–52 cm in terms of total errors.

#### 3.3.2. Estimation of GCP-Based Image Mapping Error

After localization of the 12 GCPs in the orthomosaic images and DEMs, the average errors (x, y, z, xy, and total errors) between the projected coordinates (UTM zone 52N) of the GCPs and the measured coordinates of the corresponding positions in the orthomosaic images were compared (Table 6). Examples of 12 GCP positions extracted from each orthomosaic image are shown in Figure 9b. In contrast to the three network RTK GNSS positioning results, which, on average, had relatively small errors of less than 39 cm, the three post-mission PPP processing results exhibited larger error values (approximately 102 cm or more) than the total errors obtained from the network RTK GNSS positioning results. When using traditional GNSS positioning, significant positioning errors at least approximately 304 cm resulted in the recorded total errors.

#### 3.3.3. Areal and Volumetric Differences of Mine Tailings Using Different GNSS Positioning Techniques

The methodology outlined in Figure 7 was used to examine the differences and degrees of change in the mine tailings’ 2D areas, surface areas, and volumes in the nine datasets (Table 7). The mine tailings’ 2D areas, surface areas, and volumes were calculated, and the volumetric differences were estimated using data from the network RTK GNSS No. 1; these data were set as the reference data. The 2D and surface area calculations were similar, e.g., the maximum difference was approximately 9.0 m^2^ (i.e., 0.06% of the reference 2D area) among the 2D areas, while being approximately 72.6 m^2^ (i.e., 0.42% of the reference surface area) among the surface areas in all nine cases, demonstrating that minor positioning errors had little impact when evaluating 2D areas and surface areas. However, the volumetric differences were more significant. In terms of volume, the deviations from the reference data were relatively large for both standalone GNSS positioning (up to 37.4%) and post-mission PPP processing (up to 24.1%) compared to the reference volume; however, this variation was very small, i.e., less than 0.4%, for the other two network RTK GNSS positioning datasets.

## 4. Discussion

The comparison of the results of the nine datasets revealed that the post-mission PPP processing was less accurate than the RTK GNSS method in terms of positioning accuracy. However, the error of the standalone GNSS was reduced after post-mission PPP processing. Overall, we found that RTK GNSS positioning was the most accurate of the compared positioning techniques in terms of both relative camera location error and GCP-based image mapping error. With standalone GNSS, the relative camera location error exhibited an average total error of 112.1 cm, while with RTK GNSS positioning, the error improvement rate was 98.6%, with an average total error of 1.6 cm. In addition, when post-mission PPP processing was used, the error improvement rate was 69.5%, with an average total error of 35.3 cm. For the errors estimated using the GCPs, the standalone GNSS positioning showed an average total error of 321.8 cm, and the network RTK GNSS positioning’s average total error was 31.3 cm. With post-mission PPP processing, the average total error was 131.0 cm, and the error improvement rates were 90.3% and 59.3%, respectively, for network RTK GNSS positioning and post-mission PPP positioning. For the error estimations, geographic coordinates were projected to the UTM orthogonal coordinates; this projection procedure could induce an error of about one millimeter within 3000 km of the central meridian [43]. The remaining bias, due to differences in co-ordinate frames (ITRF and epoch) for each GNSS positioning technique—for example, the network RTK using Korea Geodetic Datum 2002, which refers to ITRF2000 (epoch 2002.0) [44], and the standalone GNSS positioning using WGS84 (G2139) frame—can affect the GCP-based image mapping errors. This problem will be considered in depth in further work.

Mine tailings are a source of potential pollution [45] or reprocessing materials [46]. Using the UAV image-based estimation of the volume, we found that the network RTK GNSS positioning technique’s volumes had the smallest deviation (the absolute difference was up to 0.4% of the reference volume) compared to the reference volume from the initial data obtained using network RTK GNSS positioning. In addition, the post-mission PPP processing also exhibited relatively stable deviation (the absolute difference was up to 37.4% of the reference volume) compared to the standalone GNSS technique (the absolute difference was up to 24.1% of the reference volume) (Table 7). We applied direct georeferencing, which can be effectively utilized in mountainous or glaciated areas [47] where it is difficult to implement GCPs. However, if GCPs are applied in the orthomosaic- and DEM-generation processes, the positional accuracy can be improved [48], and, in the case of vertical bias alone, it can be corrected using a single GCP [49].

The standalone GNSS positioning showed a relatively large absolute error and deviation, as expected, and the network RTK GNSS positioning method used to improve it exhibited excellent accuracy and precision for sensing the orthomosaic images. Although the post-mission PPP processing result deviated more than the RTK result in terms of the relative camera location error and volumetric evaluation, it was sufficiently effective because the maximum total error of the post-mission PPP processing result was approximately 49.8% of the best standalone GNSS positioning error in terms of the relative camera location error, as well as approximately 79.9% of the best standalone GNSS positioning error in terms of the GCP-based image mapping error.

Unlike RTK GNSS positioning, which is used to perform highly accurate positioning via high-end equipment with internal RTK GNSS receivers, post-mission PPP processing implements a positioning technology that is simple, fast, and accessible by accessing an online cloud service, rather than requiring expensive hardware and software solutions. This fact implies that post-mission PPP processing can be applied effectively to standalone GNSS receivers that are built into common devices in various fields, and post-mission PPP processing, thus, has a comparative advantage in research and work related to UAV images that require a positioning error of a few centimeters or decimeters.

Post-mission PPP processing is currently being used in a variety of studies that involve positioning technologies, including mining deformation [50], as well as areas that lack GNSS signal correction infrastructure, e.g., polar regions [51]. However, the utilization of post-mission PPP processing is rarely attempted, other than for RTK GNSS positioning applications, particularly in UAV research. Post-mission PPP processing can be applied to UAV positioning research without requiring additional equipment, e.g., network RTK GNSS receivers and network RTK GNSS broadcasting systems or base stations, at known locations around a given study area. Thus, post-mission PPP processing is a highly effective positioning technology that is used for imaging areas with insufficient geospatial infrastructure or rugged terrain where the installation of base RTK GNSS stations is difficult.

## 5. Conclusions

In this paper, we compared applications using the three measurements of network RTK GNSS positioning, standalone GNSS positioning, and post-mission PPP processing through cloud computing to evaluate positioning accuracy, when generating topography and orthomosaic images, by obtaining aerial images of an abandoned mining area using UAVs. By conducting the accuracy evaluation, we found that the accuracy was in the ascending order of network RTK GNSS positioning, post-mission PPP processing, and standalone GNSS positioning in terms of the relative camera location error (less than 3 cm, approximately 27–52 cm, and greater than 104 cm in total error, respectively), the GCP-based orthomosaic image mapping error (less than 39 cm, approximately 102–181 cm, and greater than 227 cm in total error, respectively), and the areal and volumetric difference evaluation of the mine tailings. However, the cloud computing-based post-mission PPP processing technique demonstrated a high error improvement rate (69.5% of the total error of relative camera location and 59.3% of the total error of GCP-based orthomosaic image mapping) compared to the initial standalone GNSS positioning-based location data.

The results indicate that, in terms of restrictions on infrastructure, post-mission PPP processing can be used as an alternative to network RTK GNSS positioning for UAVs surveying areas that demand spatial accuracy of several centimeters or decimeters. In future work, post-mission PPP processing will be employed to polar regions that lack GNSS signal-correction infrastructure to assist in various mapping applications, e.g., vegetation, landform, glacier, sea ice, snow, and fauna mapping.

## Figures and Tables

**Table 1:** UAV specifications [35].

Equipment	Category	Specifications
UAV	Model	DJI Phantom 4 RTK
	Imaging sensor	20 mega pixels
	GNSS positioning accuracy in RTK mode	Vertical 1.5 cm and 1 ppm (RMS), horizontal 1 cm and 1 ppm (RMS)

**Table 2:** Specifications of GNSS equipment [40].

Equipment	Category	Specifications
Network RTK GNSS receiver	Model	Emlid Reach RS2
	Positioning accuracy in RTK mode	Vertical 1.4 cm and 1 ppm (RMS), Horizontal 0.7 cm and 1 ppm (RMS)

**Table 3:** UAV flight and image acquisition specifications.

Category	Specifications
Number of UAV image datasets	Three flights using network RTK GNSS positionings (RTK1, RTK2, and RTK3), three flights using standalone GNSS positioning (GNSS1, GNSS2, and GNSS3), and three post-mission processing datasets using PPP positioning on images with standalone GNSS positioning (PPP1, PPP2, and PPP3)
Number of images acquired per flight	77
Flight height	110 m above ground level at starting point of flights
Flight duration	4 min, 57–59 s
Forward overlap	75%
Side overlap	70%
Number of images used for image mosaicking	73

**Table 4:** GCP measurement specifications.

Category	Specifications
Number of GCPs	12
Number of measurements for averaging	5

**Table 5:** Average relative camera location error of each GNSS positioning technique.

Case	X (cm)	Y (cm)	Z (cm)	XY Error (cm)	Total Error (cm)
RTK1	0.4	0.6	2.0	0.7	2.1
RTK2	0.5	0.4	1.3	0.6	1.4
RTK3	0.4	0.3	1.2	0.5	1.3
GNSS1	20.0	25.0	117.7	32.0	122.0
GNSS2	9.8	15.2	107.9	18.1	109.4
GNSS3	15.2	21.2	101.6	26.0	104.9
PPP1	3.1	3.1	26.2	4.4	26.6
PPP2	4.1	2.3	26.8	4.8	27.2
PPP3	13.4	8.0	49.9	15.6	52.2

**Table 6:** Average geolocation error of each orthomosaic image and DEM compared to reference GCPs.

Case	X (cm)	Y (cm)	Z (cm)	XY Error (cm)	Total Error (cm)
RTK1	8.5	6.1	36.4	11.0	38.3
RTK2	5.3	4.6	34.7	7.9	36.1
RTK3	4.3	4.8	17.7	7.2	19.6
GNSS1	149.3	28.3	396.0	155.1	434.9
GNSS2	152.1	51.6	230.9	168.2	303.5
GNSS3	137.1	82.4	133.3	169.0	227.0
PPP1	81.7	35.1	155.6	89.4	181.3
PPP2	64.5	10.0	73.7	65.5	102.0
PPP3	102.0	14.6	32.2	103.5	109.8

**Table 7:** Geometric features, including 3D areas, surface areas, and volumes, of each DEM from nine datasets, as well as differences in mine tailings’ volumes compared to volume sourced from RTK1 dataset.

Case	2D Area (m^2^)	Surface Area (m^2^)	Volume (m^3^)	Difference from RTK1 Volume (%)
RTK1	16,129.5	17,071.6	52,482.4	-
RTK2	16,128.0	17,083.8	52,279.0	−0.4
RTK3	16,130.2	17,136.4	52,466.6	0.0
GNSS1	16,129.4	17,063.0	71,291.4	35.8
GNSS2	16,122.7	17,068.7	72,125.1	37.4
GNSS3	16,131.7	17,120.0	68,582.1	30.7
PPP1	16,125.6	17,041.6	57,540.4	9.6
PPP2	16,125.6	17,058.4	58,542.2	11.6
PPP3	16,128.9	17,114.2	65,139.2	24.1

## Data Availability

The datasets used in this study are available from the corresponding authors upon request.
